# Supplementary material for: Identity abuse against sexual and gender minority communities: The Being LGBTQI+ in Ireland study
Source: PLoS One. 2025 Nov 26;20(11):e0335595. doi: 10.1371/journal.pone.0335595 (PMC12654936; doi:10.1371/journal.pone.0335595)
Supplement: S1 File — (DOCX) [file pone.0335595.s001.docx]

| Title of Paper | Status | Aim of paper | Variables |
| --- | --- | --- | --- |
| Housing difficulties among participants in the XXXX study | Undergoing review | To examine the prevalence of housing difficulties among the LGBTQI+ population in Ireland and identity potential socio-demographic predictors of housing difficulties. | Experience of housing difficulties  Reasons for housing difficulties  Socio-demographic variables: Age; Gender identity; Sexual orientation; Ethnicity; Highest level of education completed; Employment status; Area living; Disability status; Family support for LGBTQI+ identity; Struggling with weekly expenses |
| Fostering school belonging: suggestions from young gender diverse learners in the XXXX study | Submitted | This paper presents findings on a sample of gender diverse 14-18 years olds’ suggestions for improving secondary schools for LGBTQI+ students in Ireland. | Open ended question on things that could be done to improve school for LGBTQI+ students. |
| Being LGBTQI+ in Ireland: The positive aspects of being a young person from within a sexual and gender minority community. | Undergoing review | This paper presents findings on a large sample of young LGBTQI+ people’s (14-25 years old) self-reported positive aspects of their identities, with a particular focus on the Irish context. | Open ended question: What do you like most about being LGBTQI+? |
| Impact of anti-LGBTQI+ hate speech in media on LGBTQI+ people’s wellbeing and mental health: The XXXX study | Submitted | This paper examines the mental health and wellbeing impact of exposure to hate speech though the media on LGBTQI+ individuals. | Ever experienced or witnessed anti-LGBTQI+ hate speech either online or in public media?  “What impact, if any, has this commentary had on you or the LGBTQI+ communities?  Wellbeing and mental health measures: Happiness, Self-esteem, Resilience, Depression, Anxiety and Stress, and Alcohol Use |
| Perspectives of trans and gender non-conforming people on general health care in the XXXX (blinded for review) study | Undergoing review | To examine trans and gender non-conforming participant’s experiences of general health care and compare their experiences to those of cisgender sexual minority participants. | 1) Accessing healthcare (Yes/No):  2) Healthcare utilisation experiences (4-p scale  3) General Health rating (5-p scale)  4) Healthcare practitioner support (5-p scale):  Open-ended question on experiences of physical or mental health services, or suggestions for improvement. |
