# Supplementary material for: Identity abuse against sexual and gender minority communities: The Being LGBTQI+ in Ireland study
Source: PLoS One. 2025 Nov 26;20(11):e0335595. doi: 10.1371/journal.pone.0335595 (PMC12654936; doi:10.1371/journal.pone.0335595)
Supplement: Attachment — Submitted filename: Response to Reviewer_29Sept2025.docx [file pone.0335595.s003.docx]

| **Reviewer’s Comments** | **Author Response** |
| --- | --- |
| Reviewer #1: This manuscript presents a technically sound, well-designed mixed-methods study using a large national cohort to examine the prevalence and predictors of identity-based abuse among LGBTQI+ populations in Ireland. The combination of quantitative analyses (chi-square tests and multivariate logistic regression with clearly defined p-values) and qualitative analysis translates to rigorous and comprehensive research with appropriate controls. The findings robustly support the authors’ conclusions regarding high prevalence rates and subgroup differences, and the manuscript is clearly structured, logically organized, and written in standard English. Only minor improvements are suggested. Overall, the study meets methodological and reporting standards and merits publication after minimal refinements as listed below: | Thank you |
| Line 62-66: A clear picture of what constitutes abuse, or at least how it is defined in this paper earlier on would be helpful. Table 2 (line 265) illustrates the different types of abuse and what they mean in the context of the survey, but I found myself wondering about the relationship between ‘abuse’ and related concepts like harassment, assault, violence, hate crime, and microaggression up until that point. The terms hate speech, harassment, assault, violence, and discrimination are used throughout the introduction, and it would be important to clarify whether all of those acts fall under the broader umbrella of abuse. | We have added the following to address the points raised:  Identity abuse is an umbrella term used to describe a range of harmful behaviours directed towards a person because of their identity. It can take the form of verbal threats, physical assault, and sexual violence [3]. Rooted in heteronormative, cisnormative, racial, ageist and ableist assumptions, identity abuse may target aspects such as gender identity, sexual identity, race, age and physical or mental ability [4]. What distinguishes identity abuse from other forms of abuse is that it stems specifically from bias, prejudice and hatred of a person’s immutable identity [5]. Examples include, hate speech - offensive material intended to degrade a particular social group [6], and microaggressions, which undermine members of marginalised groups through biased actions, whether conscious or subconscious [7]. |
| Line 74-83: A little bit of context regarding the referenced studies would be helpful. Even just including where the study was conducted (e.g. In these studies conducted in X and Y…) and how the study was conducted (e.g. survey, focus group, interview). | Have added where and how the studies were conducted. |
| Line 86-88: What ages are included under the term young people? A general comment which I come back to later is that there is a lack of defining what ‘young’ means in the paper. Sometimes they are defined as people under 18, and sometimes they are under 35. I think clearly defining distinct terms to indicate those below 18 vs. those below 35 would be important. And like the comment above, it would be helpful to have more context about the study being mentioned here. | We have clarified the ages of the participants where young was referenced, and have added more context about the study. |
| Line 97-98: Please provide a little more context about the referenced study. | Have added more study information. |
| Line 101-105: Please provide a little more context about the referenced study. | Have added more study information. |
| Line 116-118: Please provide a little more context about the referenced study. | Have added more study information. |
| Line 122-136: The rationale for including the variables are very clear, except for urbanity. Why was this included? How has urbanity affected likelihood of experiencing abuse in prior studies if there are any? | Have added the following:  US based research suggests that LGBTQ+ young people living rurally are more at risk of abuse and discrimination, with the conservative nature of rural areas being cited as well as a lack of support and resources [26-27]. While LGBTQ+ people in Ireland identify a lack of supports and services in rural communities [28], it is not clear whether it rural versus urban living impacts one’s experience of LGBTQI+ identity abuse. |
| Line 162-166: What are the print and digital media (e.g. are they newsprints, magazines), and how does digital media differ from social media? Were they advertisements? Which social media platforms were used? What does interviews with regional radio stations mean? Were the researchers interviewed by radio hosts to discuss and promote the study? More detail and clarity would be nice. | This has been clarified. |
| Line 171-203: In lines 143-145 it is mentioned that an advisory steering committee comprised of members of the sexual and gender minority community were involved in the survey design. I think this is an important point to expand on in the methodology. Involvement of the community being studied follows principles of community-based participatory research (CBPR) which increases the validity and reliability of the study. CRPR principles are also a cornerstone of minority research, and I would add a reference to support that. | We have expanded on this. |
| Line 199: Closing bracket without opening bracket. | Fixed |
| Line 201-202: In line 485 it is mentioned that there is no reliable estimate of LGBTQI+ population in Ireland. I think this is important to mention here to describe why the sample representativeness is unknown. I was left wondering why it was unknown until I got to line 485. | We have added an explanation for why sample representativeness is unknown. |
| Line 225-234: Is there a specific qualitative technique or approach that was used and could be referenced here? Thematic analysis framework developed by Braun & Clarke come to mind. Mentioning a concrete qualitative model/approach would strengthen the qualitative portion of this study. | We have expanded on the qualitative approach that was used. |
| Line 225-234: What is the role of AH in the qualitative analysis specifically? What does it mean to have a second researcher? Did they go back and review the data/codes after the discussions? Or were they just included in the discussions? Did anything change after the discussions took place and AH got involved? I have no idea what AH’s role was from the current description, and so I can’t really agree that it enhances trustworthiness of the process. I also wonder if validity and reliability are better words to use than trustworthiness. | We have clarified the analytic process and the role of the second researcher. |
| Line 237-241: Even though the specific data is available in Table 1, I find it helpful to include percentages in the sentences directly (e.g. Nearly a third (30.2%) identified as transgender and…). Percentages are included in the latter half of the paragraph, and I would encourage the same in the first half of this paragraph. | Have included the %s. |
| Line 252: Figure 1 is missing. | Apologies. Figure 1 has been uploaded |
| Line 287: Related to an earlier comment, I think there needs to be a clear distinction between young people between ages 14-18 and young people aged under 35. Earlier the sample is called ‘young’ because they were under 35. Now the same word is used to describe people who 14-18. Could terms like Underaged Youth be used for people 14-18, and Young Adult for people who are up to 35 years old? Aside from clarity, I think someone who is 14 and another person who is 35 are at vastly different stages of life and would experience have different forms and degrees of abuse. | To enhance the clarity, we’ve deleted the term young and referenced the age group instead. |
| Line 293: What does the colour green in Table 3 signify? There is no description of that. | Green signifies a statistically significant result. This has been added to the table description at the bottom. |
| Line 300-301: Could percentages be included after the number of participants in the sentence for quick context? | Have added % |
| Line 303: Does the term women in this case include just cis women and not trans women, as trans and gender non-confirming folk are grouped separately? If so I would use cis women consistently because using the term women at the exclusion of trans women doesn’t seem right. | Gender-related abuse was an issue highlighted by cisgender women. For clarity, we have changed women to cisgender women. |
| Line 370-373: The higher prevalence of abuse in this study compared to EU and prior report in Ireland is quite staggering, is there any possible explanations for why that may be? | The timeframe may account for this. It would have been more appropriate to compare to the past year rate of LGBTQI+ identity abuse and we have now changed this. |
| Line 390: I think a comma or semi-colon could be used here. | Have added a comma |
| Line 400-404: Is there a way to separate this sentence into two? This was the first instance where I had to reread a sentence couple of times to understand it fully. | Have split into two sentences |
| Line 402: The word ‘also’ is repeated. | Have deleted repeated word |
| Line 406: Is there a particular reason why the acronym TGNC wasn’t used here suddenly? | We have reverted to using the acronym. |
| Line 406-407: I think TGNC and cis women should also be compared for a fuller picture/comparison between the three groups, especially since in the next sentence cisgender people are mentioned broadly. | Thank you for your suggestion. We have considered it, and as Cis men are the reference category in the regression analysis, it wouldn’t be appropriate to mention cis women in this sentence. |
| Line 409-411: Could the word ‘that’ be replaced with ‘the’? | Yes, thank you. |
| Line 441-443: When it’s mention that most were men, does it mean cis men compared to trans men? And is the term gay used here to include those who identify as lesbians as well? The wording in this sentence is very confusing as to who is included and who is not. | The survey asked people about their sexual orientation and gender identity separately. So those who identified their sexual orientation as gay included people who identified their gender as woman, man, TGNC. By saying ‘gay participants, most of whom were men’ we are just trying to give a bit more context to the reader who might be wondering if those who identified as gay were mostly men, women, or other. I hope this makes sense, but if you think the reader could be confused, we will try to clarify further. |
| Line 444: Extra closing bracket. | Fixed |
| Line 453: Extra closing bracket. | Fixed |
| Line 468-472: I think the sentence can end after ‘ethnicity’ in line 470, and a new sentence can start from ‘however’. Also, I think it would be nice to mention other studies from Ireland or EU which have found that ethnicity leads to more or less exposure to abuse for context. | Have split the sentence into two.  Have added an EU study:  An EU survey found that LGBTIQ people with multiple intersecting identities, including ethnic or migrant backgrounds, experienced higher levels of discrimination than those without these backgrounds [5]. |
| Line 470: Extra comma after the word data? | Comma deleted |
| Line 475-490: I think the advisory steering committee should be mentioned as a strength; community participation under principles of community-based participatory research (CBPR) is known to increase validity/reliability of research. | We have added community participation in the research as a strength. |
| Line 476: Extra ‘not’. | Deleted |
| Line 478-480: What is 'more covert' abuse? What does it include and look like? Again, I think it would be important to outline what abuse means and includes/excludes in this paper earlier on. | We have provided some examples of the covert types of LGBTQI+ identity abuse which the survey did not include. |
| Line 493-496: Confused as to the difference between the terms prejudiced individuals and stigmatized minority – does this sentence mean that victims of abuse are connected to others of the same identity to receive support? Could this point be clarified? | Have clarified |
| Line 497: Why is it identity harassment and not identity abuse here? | We have changed to abuse to be consistent |
| Line 498: Seems like a word is missing after ‘counter’. | Have added missing word |
| Line 508-514: Is there a reason why intersectionality theory and minority stress theory weren’t mentioned/discussed earlier in the paper? I think it is very important to discuss them early as guiding theories and lenses for this paper. I find it odd that they are only introduced/mentioned briefly at the very end. | We had mentioned intersectionality in the introduction but have made it more explicit.  We have also referenced minority stress theory in the introduction. |
| Line 536-539: Could examples of the policies and measures be provided? It doesn’t have to be in-depth – maybe just list them (e.g. education and awareness, workplace sensitivity training, inclusive language policy, etc.). | Have added the following:  These results highlight the requirement for polices and measures to safeguard against identity abuse for LGBTQI+ people, such as robust hate crime/speech legislation, public education and awareness to combat prejudice, and inclusive policies and practices across in education, workplace and healthcare settings. |
| Line 542-546: Why is the contribution of the advisory steering committee not acknowledged? | Apologies, we have used the terms advisory steering committee and research advisory groups interchangeably. For consistency we have changed to using the term Research Advisory Group throughout and their contribution is acknowledged. |
